# Fluorescent Magnetic Bioprobes by Surface Modification of Magnetite Nanoparticles

**DOI:** 10.3390/ma6083213

**Published:** 2013-07-31

**Authors:** Paula C. Pinheiro, Ana L. Daniel-da-Silva, Daniela S. Tavares, M. Pilar Calatayud, Gerardo F. Goya, Tito Trindade

**Affiliations:** 1 Department of Chemistry-CICECO (Centre for Research in Ceramics and Composite Materials), Aveiro Institute of Nanotechnology, University of Aveiro, 3810-193 Aveiro, Portugal; E-Mails: pcpinheiro@ua.pt (P.C.P.); ana.luisa@ua.pt (A.L.D.S.); danielatavares@ua.pt (D.S.T.); 2 Institute of Nanoscience of Aragón (INA), University of Zaragoza, 50018 Zaragoza, Spain; E-Mails: pilarcs@unizar.es (M.P.C.); goya@unizar.es (G.F.G.)

**Keywords:** magnetic nanoparticles, fluorescence, polyelectrolyte, surface functionalization

## Abstract

Bimodal nanoprobes comprising both magnetic and optical functionalities have been prepared via a sequential two-step process. Firstly, magnetite nanoparticles (MNPs) with well-defined cubic shape and an average dimension of 80 nm were produced by hydrolysis of iron sulfate and were then surface modified with silica shells by using the sol-gel method. The Fe_3_O_4_@SiO_2_ particles were then functionalized with the fluorophore, fluorescein isothiocyanate (FITC), mediated by assembled shells of the cationic polyelectrolyte, polyethyleneimine (PEI). The Fe_3_O_4_ functionalized particles were then preliminary evaluated as fluorescent and magnetic probes by performing studies in which neuroblast cells have been contacted with these nanomaterials.

## 1. Introduction

Magnetic nanomaterials offer a myriad of attractive possibilities in nanomedicine, including cell separation, biotracking procedures, site-specific drug targeting, delivery and controlled release, and medical imaging (MRI) [1,2,3,4,5]. Most of these applications require the nanoparticles to be chemically stable and well dispersed in physiological medium. It has been demonstrated that adequate coating of magnetic nanoparticles enhances the colloidal stability, preventing their aggregation in solution and improving their chemical stability [3,6]. Silica has been used very often as the surface coating material for inorganic nanoparticles, taking advantage of being biocompatible and hydrophilic and, also, because the surface silanol groups can easily react with organic molecules to provide specific functionalities [7,8]. Additionally, surface modification of silica coated magnetite nanoparticles with polyelectrolytes can enhance their stability against flocculation in aqueous media, due to electrostatic repulsive forces [9].

The assembly of building blocks with different functionalities can provide multimodal platforms for better efficiency and to facilitate their applications in different fields, such as bioscience and medicine. In particular, magnetic nanomaterials have been investigated for multifunctional vectors for* in vitro* clinical diagnosis by adequate chemical surface functionalization with fluorophores [10,11,12]. This strategy has been reported for a variety of systems, including the use of magnetite nanoparticles carrying fluorescent properties and other functionalities that confer the ability to drive the particles into localized targets [9,13,14]. The development of magnetic nanosized vectors is of great relevance in a number of biomedical applications, such as drug delivery, optical biotracking and optical-assisted magnetic bioseparation. Thereby, a number of methods have been reported in the literature aiming at modifying the surface of iron oxides, most often maghemite and magnetite, with fluorescent compounds. Challenges remain in their fabrication, which frequently involve multi-step reactions to prevent the quenching of the fluorophore. Table 1 summarizes illustrative approaches on the use of silica coated magnetite nanoparticles that have been investigated in this specific context.

**Table 1:** Surface modification strategies of silica coated magnetite with fluorescent labels.

Surface coating	Fluorophore	Potential bioapplications	Reference
None	FITC	MRI	[14]
None	FITC	MRI, cell tracking and drug delivery	[15]
None	QDs	Cell tracking and drug delivery	[16]
PNIPAM	None	Drug delivery	[17]
PEG	FITC	Drug delivery	[18]
PS	Pyrene	Bio-separation	[19]
PDADMAC/PSS	QDs	Cell tracking	[20]
APTS	Protoporphyrin IX	*In vitro* biological image	[21]

FITC: fluorescein isothiocyanate; QDs: quantum dots; PNIPAM: poly(N-isopropylacrylamide); PEG: poly(ethylene glycol); PS: poly(styrene); PDADMAC: poly(diallyldimethylammonium chloride); PSS: poly(sodium styrene sulfonate); APTS: 3-aminopropyltriethoxysilane.

**Figure 1:**
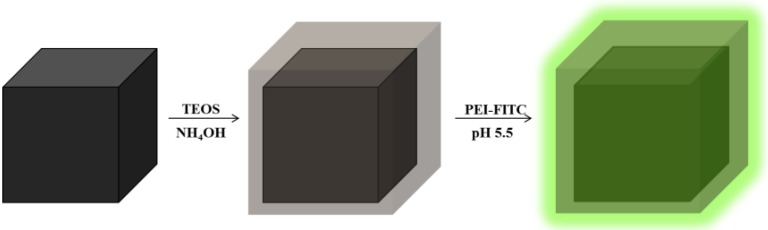
Scheme for the surface modification of Fe_3_O_4_ nanoparticles.

The aim of this research was to develop an alternative simple method for the functionalization of Fe_3_O_4_ particles with FITC to afford fluorescent magnetic vectors that can be used in bioassays. The general scheme of this multi-step functionalization involved the synthesis of Fe_3_O_4_ nanoparticles, by precipitation of an iron (II) salt (FeSO_4_) in alkaline medium (KOH), and subsequent coating with amorphous SiO_2 _shells (Figure 1). The surfaces of the core/shell particles were then modified by treatment of the colloids with a polyelectrolyte (PEI) that has been previously labeled with fluorescein isothiocyanate (FITC), thus conferring fluorescence to the resulting material. The potential application of these nanomaterials as optical and magnetic markers for* in vitro* cell internalization will be discussed.

## 2. Experimental

### 2.1. Chemicals

Ferrous sulfate heptahydrate (FeSO_4_·7H_2_O) (>99%) and ethanol (CH_3_CH_2_OH) (>99%) were obtained from Panreac (Spain). Potassium nitrate (KNO_3_) (>99%), tetraethyl orthosilicate (Si(OC_2_H_5_)_4_, tetraethyl orthosilicate (TEOS)) (>99%) and branched polyethyleneimine (PEI, M_w_ = 25,000) were purchased from Sigma–Aldrich. Potassium hydroxide (KOH) (>86%) was purchased from Pronolab (Portugal). Ammonia solution (25% NH_3_) was obtained from Riedel-de-Häen (Germany). All chemicals were used as received without any further purification.

### 2.2. Synthesis of Magnetite (Fe_3_O_4_) and Silica Coated Magnetite (Fe_3_O_4_@SiO_2_)

Magnetite nanoparticles were synthesized by hydrolysis of FeSO_4_·7H_2_O under a N_2_ stream [22,23]. In a typical synthesis, a mixture of KNO_3_ (0.016 mol, 1.62 g) and KOH (0.200 mol, 11.23 g) in 60 mL of ultrapure water, previously deoxygenated, was prepared under N_2_ and, then, was added dropwise to the Fe (II) solution (0.5 M, 20.0 g), under N_2_ and at 90 °C. The black precipitate formed was stirred over 1 h at 90 °C, left overnight and, then, washed thoroughly with deionized water. Silica coating of magnetite nanoparticles was performed via the alkaline hydrolysis of TEOS. First, Fe_3_O_4_ nanoparticles (200 mg) were dispersed in ethanol (150 mL) and kept immersed in an ice bath, over 15 min under sonication (horn Sonics, Vibracell). Then ammonia solution (12 mL) and TEOS (400 μL) were slowly added to the Fe_3_O_4 _suspension, and the mixture was sonicated in an ice bath for 2 h; volume ratio of ammonia: TEOS was kept to 30:1. Finally, the SiO_2_ coated particles were collected magnetically using an NdFeB magnet, and the isolated powders were washed thoroughly with ethanol.

### 2.3. Surface Functionalization of Fe_3_O_4_@ SiO_2_ with Fluorophore FITC

In order to functionalize the magnetite particles with FITC, the fluorophore was first covalently bound to the PEI chains by reacting the isothiocyanate group of FITC with the primary amine groups of PEI [24]. Thus, 312.6 mg (0.013 mol) of PEI were dissolved in 30 mL of ultrapure water, and this solution was mixed with 12.6 mg (0.03 mmol) of FITC. The pH of the solution was adjusted at 11.0 by using 0.01 M NaOH solution. The mixture reacted overnight in dark conditions, at room temperature under mechanical stirring. The resulting FITC-labeled PEI (termed here as PF) was purified by dialysis, until no fluorescence was detected in the dialysate, under excitation at 494 nm. Bare Fe_3_O_4 _particles and SiO_2_ coated Fe_3_O_4_ nanoparticles were then coated with PF (samples designated by Fe_3_O_4_-PF and Fe_3_O_4_@SiO_2_-PF, respectively). In a typical procedure, 10 mg of nanoparticles were added to the PF solution (10 mL) at pH 5.5 and stirred for 1 h. Then, the suspended solid was collected magnetically and washed thrice with distilled water to remove the excess PF.

### 2.4. Characterization of the Nanoparticles

Fourier transform infrared (FTIR) spectra of the nanoparticles were collected using a spectrometer Bruker optics tensor 27 coupled to a horizontal attenuated total reflectance (ATR) cell, using 256 scans at a resolution of 4 cm^−1^. The crystallite phase of the magnetite nanoparticles was identified by X-ray diffraction (XRD). The X-ray powder diffraction pattern of the magnetic core was recorded using an X-ray diffractometer Philips X’Pert equipped with a Cu*Kα* monochromatic radiation source. The MNPs average size, distribution and morphology were analyzed by transmission electron microscopy (TEM) using an FEI Tecnai T20 microscope and operating at 200 keV. High resolution transmission electron microscopic (HR-TEM) images were obtained by using an FEI Tecnai F30 microscope operated at an acceleration voltage of 300 KV. Samples for TEM analysis were prepared by evaporating dilute suspensions of the nanoparticles on a copper grid coated with an amorphous carbon film. The zeta potential measurements were performed by using a Zetasizer Nanoseries instrument from Malvern Instruments. The zeta potential measurements were performed in samples prepared with distilled water and analyzed immediately after vigorous stirring. Magnetization of the samples was recorded as a function of the applied magnetic field, at 300 K. All measurements were performed on a vibrating sample magnetometer (VSM) ADE technologies model EV7. The fluorescence experiments were performed using a fluorometer FluoroMax3-HORIBA Jobin Yvon, using quartz cuvettes.

### 2.5. Interaction of Magnetic Nanostructures with Living Cells

Cell line: Human neuroblast SH-SY5Y cells (ATCC CRL-2266) were cultured in Dulbecco’s modified Eagle’s medium (DMEM) and Ham’s F12 (1:1) with 15% fetal bovine serum, 100 IU/mL penicillin, 100 μg/mL streptomycin and 2 mM l-glutamine. Cells were maintained at 37 °C in a saturated humidity atmosphere containing 95% air and 5% CO_2_. For experiments, cells were seeded in 6-well plates at a density of 75,000 cells/mL and incubated 24 h prior to experiments. The* in vitro* experiments were designed at 10 µg/mL concentrations of ferromagnetic nanostructures and 24 h of incubation. After the incubation time, the cells were washed, and the modified-DMEM was replaced with ordinary DMEM. Control experiments were performed with growth medium without nanoparticles.

Optical microscopy: the visualization of the cells was carried out using the inverted microscope, Nikon Eclipse TE2000*-*S.

Confocal microscopy: the incorporation of Fe_3_O_4_@SiO_2_-PF into SHSY5Y cells was studied using an Olympus IX81 inverted microscope. The cells were incubated with fluorescent Fe_3_O_4_@SiO_2_-PF. One control experiment was carried out in order to calibrate against possible autofluorescence of the cells.

## 3. Results and Discussion

**Figure 2:**
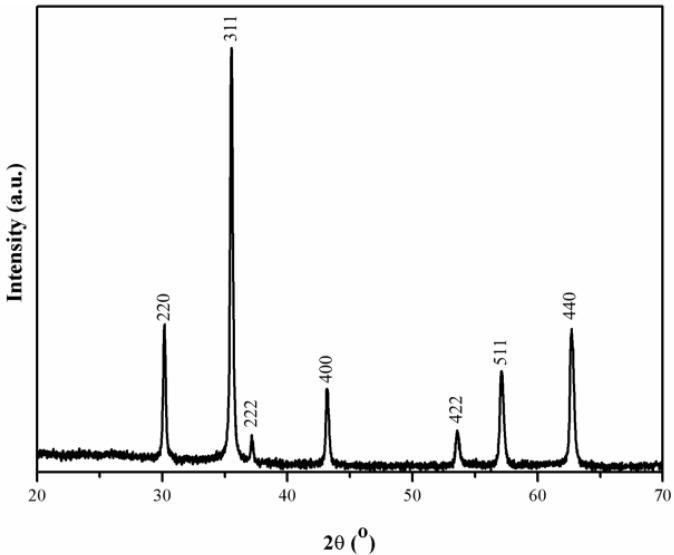
XRD diffraction patterns of the magnetite powders.

**Figure 3:**
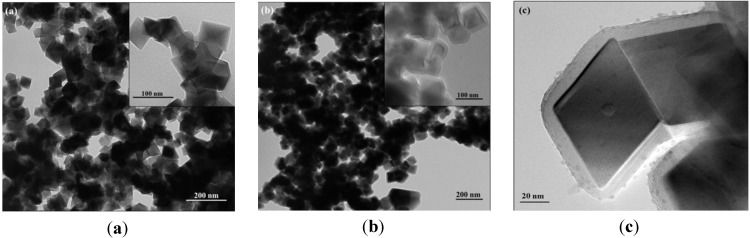
TEM images for magnetite (**a**); and silica coated magnetite (**b**,**c**) particles.

The first step of this work involved the synthesis of the magnetic cores and their encapsulation in amorphous SiO_2_ shells through the hydrolysis and condensation of tetraethyl orthosilicate (TEOS) in the presence of the magnetic nanoparticles. It has been reported that the method of hydrolysis of FeSO_4_ as employed in this research results in magnetic powders composed of magnetite as the main crystalline phase [22]. Figure 2 shows the powder XRD patterns for the sample obtained here, which are consistent with the presence of the inverse spinel structure of magnetite (JCPDS file no. 19-0629). The morphology and size distribution of the particles were examined by transmission electron microscopy. Figure 3 shows the TEM images of the magnetic particles before and after SiO_2_ coating. These results indicate that the Fe_3_O_4_ particles have a well-defined cubic shape and an average dimension of 80 nm (diagonal used as parameter). The growth of amorphous SiO_2_ shells onto the Fe_3_O_4_ particles was also confirmed by TEM observations. Indeed, Figure 3b shows two regions with different contrast indicating that the cubic Fe_3_O_4_ particles were successfully coated with a thin layer of a different phase. Magnified TEM images (Figure 3c) of selected coated particles reveal a thin coating of about 8 nm thickness of an amorphous material, as expected for the presence of SiO_2 _obtained via sol-gel method [22]. Note that the high-magnified image of the silica shells (Figure 3c) shows discrete spheroidal nanoparticles (sized smaller than 5 nm) that presumably remained as vestiges of the initial SiO_2 _sol formed during the coating process.

**Figure 4:**
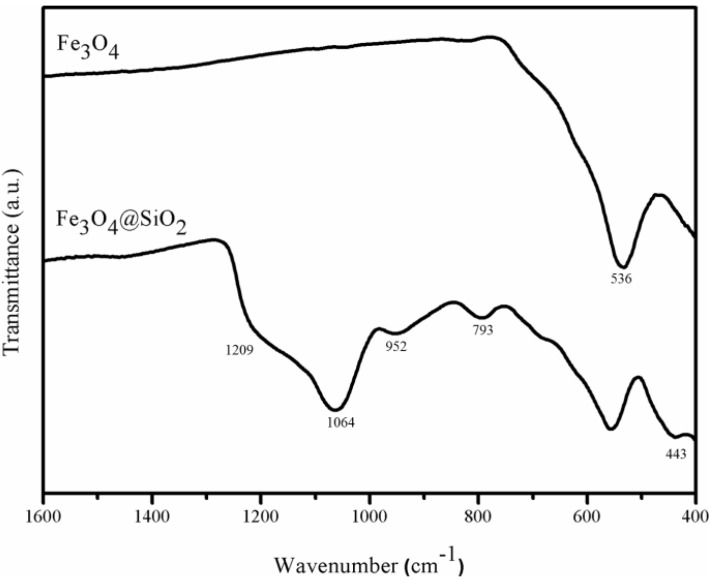
ATR-FTIR spectra of magnetite nanoparticles (Fe_3_O_4_) and silica coated magnetite nanoparticles (Fe_3_O_4_@SiO_2_).

Additional evidence for the chemical composition of the coating was obtained by ATR-FTIR spectroscopy (Figure 4). The FTIR spectrum of bare Fe_3_O_4_ nanoparticles shows a strong and broad band peaked at 536 cm^−1^, which is characteristic for this iron oxide [22], and it is also observed in the spectrum of the coated sample. The ATR-FTIR spectrum of Fe_3_O_4_@SiO_2_ (Figure 4) displays a strong broad band at 1064 cm^−1^ with a shoulder at 1209 cm^−1^, which are assigned to the υ_as_(Si–O–Si) vibration. The weaker bands at 793 and 952 cm^−1^ correspond, respectively, to the υ_s_(Si–O–Si) and υ(Si–OH) stretching modes, and the band at 443 cm^−1^ is assigned to the Si–O–Si bending, overall confirming the presence of SiO_2_ in the sample.

**Figure 5:**
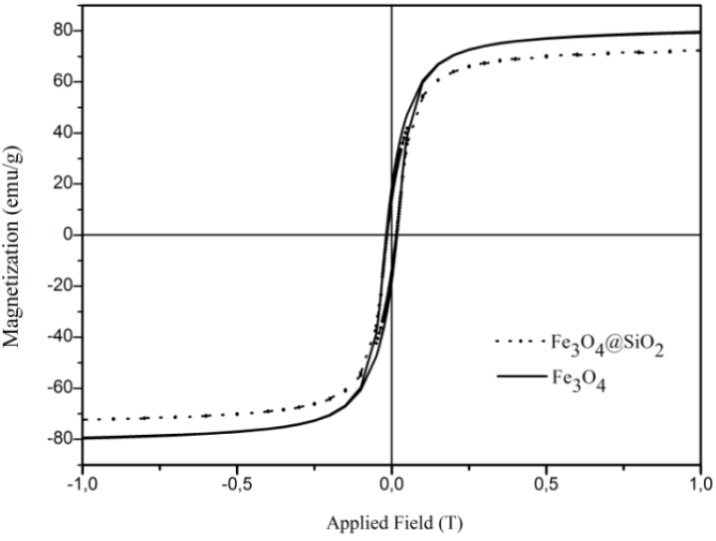
Magnetization of Fe_3_O_4_ and Fe_3_O_4_@SiO_2_ as a function of the magnetic field measured at 273 K in a diamagnetic sample holder.

The magnetization hysteresis loops of Fe_3_O_4_ and Fe_3_O_4_@SiO_2_ particles were measured at room temperature and are shown in Figure 5. As expected, both samples exhibited ferromagnetic behavior, but the uncoated particles showed higher values of magnetization saturation (80.6 emu/g) than the SiO_2_ coated samples (73.7 emu/g). The decrease of the magnetization saturation after surface coating confirms the presence of a diamagnetic material (SiO_2_) at the surface of the Fe_3_O_4_ particles [8,22].

**Figure 6:**
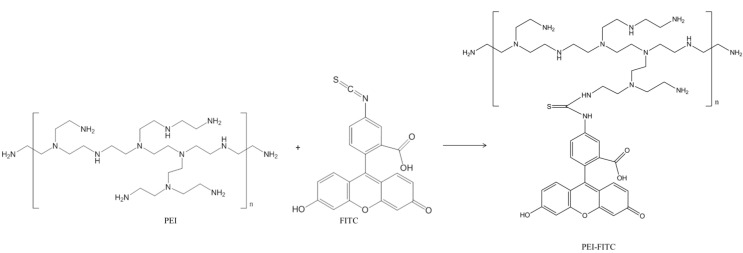
Scheme of a possible chemical path for the functionalization of polyethyleneimine (PEI) with the fluorophore FITC.

In the second stage, the surface of silica coated magnetic particles was functionalized with a fluorescent organic dye (FITC). Prior to surface modification, the FITC was covalently attached to the PEI macromolecules via formation of a thiourea bond as result of the reaction between amine groups of PEI chains and isothiocyanate group of FITC (Figure 6) [24,25,26,27]. The infrared spectrum of the collected PF conjugate did not show the broad band previously observed in the spectrum of pure FITC and that has been assigned to the stretching mode of the reactive N–C=S group (2038 cm^−1^) [25]. This is a first evidence for the formation of the thiourea bond between the dye and the polyelectrolyte PEI. Additionally, in comparison to the ATR-FTIR spectrum of PEI, the corresponding spectrum of sample PF has also shown new bands at 1508 cm^−1^ (C=C stretching), 1209 cm^−1^ (C–O–C stretching) and 1170 cm^−1^ (=C–H bending) of the aromatic system of FITC, which is consistent with the presence of this dye linked to the PEI chains [28,29].

**Figure 7:**
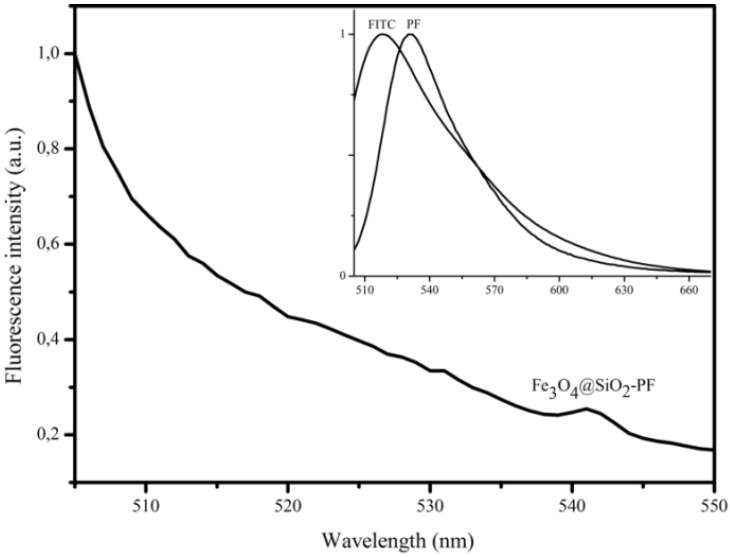
Normalized fluorescence spectra of FITC, PEI conjugated with FITC (PF) and functionalized Fe_3_O_4_@SiO_2_ with PEI-FITC (Fe_3_O_4_@SiO_2_-PF) (λ_exc_ = 494 nm).

Figure 7 (inset) shows the fluorescence emission spectra of FITC (λ_máx_ = 519 nm) and PF (λ_máx_ = 535 nm) aqueous solutions. The shape and position of the PF emission peak are consistent with those of fluorophore. The red shift observed for the PF conjugate (∆λ = 16 nm) may be due to changes in the environment of dye molecules and to interactions between neighboring molecules, which decrease their excited state energy and produce a red shift in the spectra [30]. The interactions between dye molecules may also lead to self-quenching, resulting in a reduction of fluorescence intensity.

After functionalization, the particles were easily collected by magnetic separation from the reacting suspension using an NdFeB magnet, thus attesting to their magnetic behavior. Fluorescence measurements performed on magnetite particles directly functionalized with PF revealed total quenching of the FITC emission, due to the proximity of the iron oxide surfaces. This behavior is in agreement with other reports and has been explained by a possible energy transfer process, due to the contact of fluorophore with the surface of metal oxide particles [4,31]. Figure 7 shows that the quenching was attenuated by encapsulating the magnetic nanoparticles with a silica shell prior to the introduction of the fluorescent molecule. A 6 nm red shift was observed in the fluorescence intensity maximum of the Fe_3_O_4_@SiO_2_-PF particles in relation to PF conjugate. The red shift has been previously reported on FITC incorporated in colloidal silica spheres and was attributed to changes of the environment of the fluorescence dyes [32,33]. In addition, the low fluorescence intensity may be attributed to quenching between the closed packed fluorophore molecules [4]. Due to their fluorescent properties, Fe_3_O_4_@SiO_2_-PF particles were selected to proceed with preliminary bioassays in order to evaluate their interaction with SHSY5Y cells.

**Figure 8:**
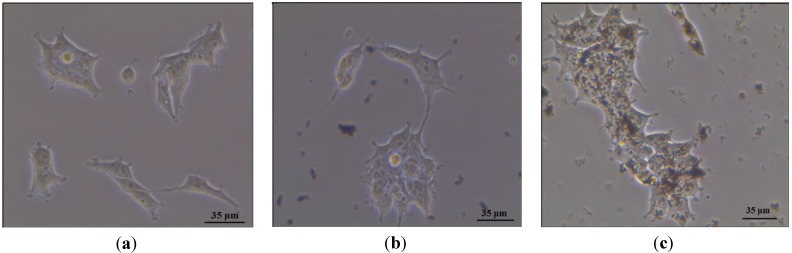
Optical microscopy images of neuroblast cells prior to interaction with ferromagnetic nanoparticles (**a**); after contact with Fe_3_O_4_@SiO_2_ (**b**); and after contact with Fe_3_O_4_@SiO_2_-PF (**c**).

**Figure 9:**
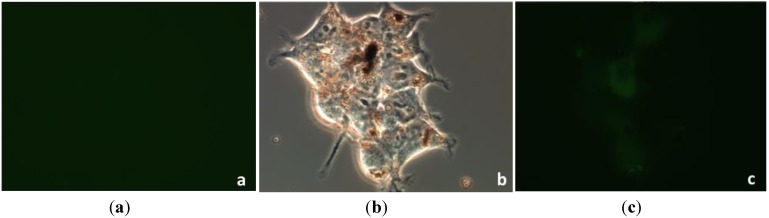
Optical microscopy images of neuroblast cells incubated with Fe_3_O_4_@SiO_2_-PF particles: (**a**) control sample in fluorescence mode; (**b**) light transmission mode, 60×; and (**c**) fluorescence mode, 60×.

*In vitro* experiments were made to evaluate the interaction of the Fe_3_O_4_@SiO_2_-PF nanoparticles with cells regarding the cell internalization process. The neuroblastoma SHSY5Y cell line was used as the* in vitro* model. The first evidence for the relevance of the surface modification proposed here to promote the interaction between the Fe_3_O_4_ nanoparticles and the cells was obtained by optical microscopy. Figure 8 shows optical micrographs of cells that have been incubated with Fe_3_O_4_@SiO_2_ and Fe_3_O_4_@SiO_2_-PF particles. For the sake of comparison, cells grown in absence of particles were also analyzed (Figure 8a). The optical images in Figure 8b,c show the microscopy results for cells after contact with Fe_3_O_4_@SiO_2_ and Fe_3_O_4_@SiO_2_-PF particles, respectively. Figure 8b shows areas containing the cells and some micrometric aggregates, but there is no evidence of interaction between cells and Fe_3_O_4_@SiO_2 _particles. Conversely, the presence of micrometric aggregates attached to the surface of neuroblasts cells suggests that Fe_3_O_4_@SiO_2_-PF were successfully targeted to the cell membrane. These results show that the interaction of the particles with these cells is favored after modification of the silica surfaces with the PF. The internalization of the surface modified Fe_3_O_4_@SiO_2_-PF particles in the cells was further confirmed by taking advantage of the presence of the fluorophore, FITC. As shown in Figure 9, the green fluorescence emission observed in Figure 9c can only be attributed to the presence of FITC at the surface of Fe_3_O_4_@SiO_2 _particles, which are observed as aggregates in the optical image acquired in the transmission mode (Figure 9b). It should be noted that not all the surface modified magnetite particles observed as aggregates in the transmission optical mode led to the green fluorescence observed in Figure 9c. This can be a consequence of the aggregation state of the particles or, simply, due to a lesser amount of FITC present at the surfaces.

**Figure 10:**
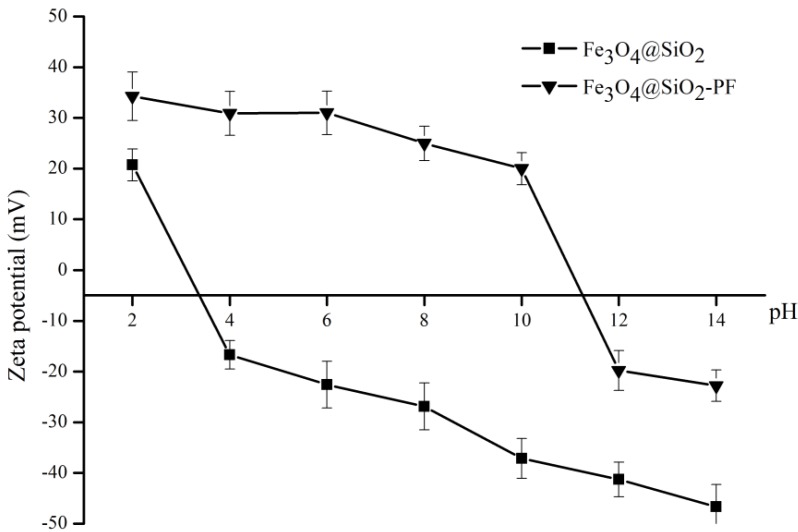
Zeta potential as a function of pH for Fe_3_O_4_@SiO_2_ and Fe_3_O_4_@SiO_2_-PF nanoparticles.

A possible explanation for the observations described above, namely the internalization of surface modified Fe_3_O_4_@SiO_2_-PF particles in the neuroblasts cells, relies on the contribution of electrostatic interactions. Figure 10 shows the zeta potential of Fe_3_O_4_@SiO_2_ and Fe_3_O_4_@SiO_2_-PF aqueous suspensions, at a pH varying from 2 to 14. At physiological pH, these cells are negatively charged, and Fe_3_O_4_@SiO_2_-PF particles are positively charged. In principle, strong attachment of the particles to the cells is expected in this case. The Fe_3_O_4_@SiO_2_-PF colloidal particles are positively charged, due to the adsorption of PEI via strong electrostatic interactions at the SiO_2_ surfaces. The literature value for pK_a_ of the most basic (primary) amine of PEI is 9.5 [34], and therefore, its zeta potential is negative only at higher solution pH values. The surface of PF-modified particles is positively charged over a wider pH range, and the isoelectric point was found to be 11.3. On the contrary, the surface of Fe_3_O_4_@SiO_2 _particles is negatively charged at pH values above 3.4. Although in both cases, the zeta potential decreased as the pH increased, the Fe_3_O_4_@SiO_2_-PF particles are positively surface charged at physiological pH, while the Fe_3_O_4_@SiO_2 _particles are negatively charged [35,36].

## 4. Conclusions

We have reported a facile strategy for the synthesis of bimodal magnetic and fluorescence nanoparticles. Firstly, cubic magnetite nanoparticles were obtained by hydrolysis of iron sulfate, and their surfaces were modified with silica shells. The fluorescent magnetite nanoparticles were obtained through attachment of a polyelectrolyte modified with the fluorophore, FITC. The colloidal stability of the ensuing hybrid system is limited, but future studies on similar systems with smaller average sizes might bring improvements in this respect. Although the optimization of this method was beyond the scope of this work, we are confident that such efforts will improve fluorescence bioimaging using the Fe_3_O_4_@SiO_2_-PF particles. Indeed, preliminary essays using these hybrid nanostructures for* in vitro* cell internalization suggests their potential as multimodal bioprobes.
